# Clinical features and prediction of long-term survival after surgery for perihilar cholangiocarcinoma

**DOI:** 10.1371/journal.pone.0304838

**Published:** 2024-07-01

**Authors:** Anna Mantas, Carlos Constantin Otto, Pim B. Olthof, Daniel Heise, Dieter Paul Hoyer, Philipp Bruners, Maxim Dewulf, Sven Arke Lang, Tom Florian Ulmer, Ulf Peter Neumann, Jan Bednarsch

**Affiliations:** 1 Department of Surgery and Transplantation, University Hospital Essen, Essen, Germany; 2 Department of Surgery and Transplantation, University Hospital RWTH Aachen, Aachen, Germany; 3 Department of Surgery, Erasmus Medical Center, Rotterdam, The Netherlands; 4 Department of Surgery, Amsterdam UMC, Amsterdam, The Netherlands; 5 Department of Surgery, University Medical Center Groningen, Groningen, The Netherlands; 6 Department of Diagnostic and Interventional Radiology, University Hospital RWTH Aachen, Aachen, Germany; 7 Department of Surgery, Maastricht University Medical Center (MUMC), Maastricht, The Netherlands; Jichi Medical University: Jichi Ika Daigaku, JAPAN

## Abstract

**Introduction:**

The treatment of perihilar Cholangiocarcinoma (pCCA) poses specific challenges not only due to its high perioperative complication rates but also due its dismal long-term prognosis with only a few long-term survivors (LTS) among the patients. Therefore, in this analysis characteristics and predictors of LTS in pCCA patients are investigated.

**Material and methods:**

In this single center analysis, patients undergoing curative-intent liver resection for pCCA between 2010 and 2022 were categorized into long-term and short-term survivors (STS) excluding perioperative mortality. Binary logistic regression was used to determine key differences between the groups and to develop a prognostic composite variable. This composite variable was subsequently tested in the whole cohort of surgically treated pCCA patients using Cox Regression analysis for cancer-specific survival (CSS).

**Results:**

Within a cohort of 209 individuals, 27 patients were identified as LTS (median CSS = 125 months) and 55 patients as STS (median CSS = 16 months). Multivariable analysis identified preoperative portal vein infiltration (OR = 5.85, p = 0.018) and intraoperative packed red blood cell (PRBC) transfusions (OR = 10.29, p = 0.002) as key differences between the groups. A prognostic composite variable based on these two features was created and transferred into a Cox regression model of the whole cohort. Here, the composite variable (HR = 0.35, p<0.001), lymph node metastases (HR = 2.15, p = 0.001) and postoperative complications (HR = 3.06, p<0.001) were identified as independent predictors of CSS.

**Conclusion:**

Long-term survival after surgery for pCCA is possible and is strongly negatively associated with preoperative portal vein infiltration and intraoperative PRBC transfusion. As these variables are part of preoperative staging or can be modulated by intraoperative technique, the proposed prognostic composite variable can easily be transferred into clinical management to predict the oncological outcome of patients undergoing surgery for pCCA.

## Introduction

Cholangiocarcinoma (CCA) is a highly aggressive malignant tumor of the biliary tract and due to its biology, non-specific clinical presentation and therefore usually late diagnosis associated with dismal clinical and oncological outcomes [1]. According to their location along the biliary tract, CCAs are categorized into different subtypes, with perihilar CCA (pCCA) being the most common, representing approximately 50–67% of the cases [2].

To this day, extended surgical resection with hilar lymphadenectomy remains the only curative-intent treatment option for patients with localized CCAs [3, 4]. Available data shows that approximately 10–40% of all patients diagnosed with CCA are considered resectable upon diagnosis [5]. Moreover, for patients with pCCA, a 90-day mortality of up to 15% after surgical treatment has been reported [6–8]. As only a minority of patients diagnosed with pCCA is eligible for curative-intent surgery and treatment-associated morbidity rates are high, it is of utmost importance to identify patients with promising long-term survival prior to actual surgery [9, 10]. Investigating long-term survival in patients with pCCA is gaining increased attention. A retrospective multicenter study published in 2019 shows a 10-year overall survival of only 13%, a median overall survival (OS) of 29.9 months and a 10-year recurrence-free survival (RFS) of 5% after surgery. The following variables have been identified as predictive factors for a dismal postoperative prognosis: high age, arterial involvement, R1 status, advanced Bismuth classification and lymph node metastases) [11, 12]. In addition, perioperative blood transfusion, low preoperative serum albumin and lymphovascular invasion (LVI) have been identified as independent predictors of adverse oncological outcome for patients with pCCA [13–15].

As data on long-term survival and its predictors is sparse in pCCA, we aimed to characterize long-term survivors (LTS) and investigate predictors of survival in pCCA patients.

## Material and methods

### Patients

All consecutive patients undergoing surgical resection for pCCA at the University Hospital RWTH Aachen (UH-RWTH) between 2010 and 2022 were included in this study. The study was conducted in accordance with the requirements of the Institutional Review Board of the RWTH-Aachen University (EK 23–289), the current version of the Declaration of Helsinki, and good clinical practice guidelines (ICH-GCP). The protocol (No. EK 23–289) has received approval from the Institutional Review Board of RWTH-Aachen University. Informed consent was waived due to the retrospective study design and collection of readily available clinical data. All clinical data, which were pseudonymized for analysis, were gathered from an institutional hepatobiliary database and analyzed retrospectively. The data were accessed for research purposes on September 30, 2023.

### Staging, preoperative work-up and surgical technique

All patients referred to our institution for curative-intent surgery for pCCA underwent a thorough diagnostic and clinical work-up as outlined in prior descriptions [10, 16]. To summarize, multiphase computed tomography (CT) scans were performed to exclude distant metastases. To evaluate the tumor extent along the biliary tract, endoscopic retrograde cholangiopancreatography (ERCP) or magnetic resonance cholangiopancreatography (MRCP) were conducted. To precisely assess the vascular anatomy including the potential invasion of major vessels at the liver hilum, multiphase CT was evaluated by a dedicated radiologist (P.B.). Criteria for portal vein involvement were mainly detection of a present or absent fat plane around the PV, evaluation of the contacting points forming a convexity or concavity between the tumor and vessel or occlusion of the PV through the tumor as previously described [17–20].

In case of pronounced cholestasis and/or persisting cholangitis, uni- or bilateral endoscopic biliary drainage was conducted preoperatively. When the estimated future liver remnant (FLR) was considered insufficient, a portal vein embolization (PVE) was performed 2 to 4 weeks prior to surgery. The indication for primary surgery was made by a team of highly experienced hepatobiliary surgeons, taking into consideration the patients’ performance status (ECOG), ASA classification and furthermore laboratory parameters displaying quantitative liver function and both LiMAx (maximum liver function capacity) testing and FLR estimation calculated on CT or MRI basis. In all cases, the decision for primary curative-intent surgery was approved by the local interdisciplinary tumor board. The standardized intraoperative surgical strategy has been described earlier [15, 21]. Portal vein resection and reconstruction was carried out individually based on localized involvement of the portal vein. For parenchymal dissection Cavitron Ultrasonic Surgical Aspirator (CUSA) was used. All reported transfusion data of fresh frozen plasma (FFP) and packed red blood cells (PRBC) refer to intraoperative application. During parenchmya transection, low central venous pressure was adhered to and, if required, Pringle maneuver conducted. Lymphadenectomy of the pericholedochal, periportal, posterior pancreaticoduodenal, the common hepatic lymph nodes and the celiac trunc lymph nodes was routinely performed. Histological analyses of the surgical samples were carried out by an experienced board-certified staff pathologist using standard H&E slides to assess pathological features (e.g. nodal status, resection margin, tumor grading or perineural invasion (PNI) defined as cancer cells infiltrating the perineural layer nerves [22, 23]). Tumor classification was performed in accordance with the current 8^th^ edition of the Union for international cancer control (UICC) staging system.

### Follow-up

From 2010 to 2017, adjuvant therapy was administered in high-risk tumor features such as positive nodal status or R1 resection. From 2017 on, it was proposed to every patient, after publication of the BILCAP trial [24]. Each patient underwent follow-up, consisting of clinical examinations, laboratory tests including tumor markers (CA 19–9) and regular CT scans. In case of suspicion of recurrent disease, additional imaging and/or biopsy of the tumor recurrence was performed.

### Statistical analysis

Cancer-specific survival (CSS) was defined as the time between the date of surgery until the date of the patients’ death caused by the tumor, or the date of the last contact if the patient was still alive. Deaths that were caused by other events than from the pCCA were censored at the time of death. RFS was defined as the duration from the date of surgery until the date of first recurrence. Patients without tumor recurrence were censored at the last follow-up or at the time of death.

Based on CSS and RFS characteristics, a LTS and short-term survivors (STS) subgroup were created respectively and compared by means of univariate and multivariate binary logistic regression to determine key differences. Based on the multivariate results a discriminable composite variable was defined. The associations of CSS and RFS with clinicopathological characteristics and the composite variable variable were assessed using univariate and multivariable Cox regression analyses in a forward selection model. Survival curves were generated by the Kaplan-Meier method and compared with the log-rank test. Median follow up was accessed with the reverse Kaplan-Meier method. All survival analyses included cases with perioperative mortality. Patients with perioperative mortality were not included in the RFS analysis. The level of significance was set to p < 0.05 and p-values were given for two-sided testing. Analyses were performed using SPSS Statistics 24 (IBM Corp., Armonk, NY, USA).

### Definition of long-term survival and poor oncological outcome subgroups

To determine characteristics of patients showing long-term survival, patients from the overall cohort were selected based on survival and recurrence patterns. To ascertain oncological characteristics, patients with perioperative mortality were excluded from the cohort. Patients with a follow up of more than 5 years and survival of more than 5 years without tumor recurrence within the first 5 years after surgery were defined as long term survivors. Patients deceased within 3 years postoperatively due to tumor recurrence within the first 2.5 years after surgery were defined as poor oncological outcome group.

## Results

### Patient cohort

Data of 209 patients who underwent curative-intent surgery for pCCA at our hepatobiliary center between 2010 and 2022 were included in this study. The patient cohort consisted of 139 men (66.5%) and 70 women (33.5%) with a median age of 68 years. Most patients presented with Bismuth Type III (53.6%, 112/209) or IV (27.3%, 57/209) tumors and were assessed as ASA III or higher (62.2%, 130/209). Radiologic portal vein invasion was present in 43.8% (91/208) and arterial infiltration in 18.7% (39/208) of cases. Pathological confirmed PVI was present in 33.8% (70/209) cases. A small subset of patients underwent neoadjuvant therapy (4.8%, 10/209). The median CA 19–9 of the whole cohort was 98.5 kU/l (range: 0–38.092 kU/l). Arterial resection was performed in 7.7% (16/209) of cases, portal vein resection in 80.9% (169/209) as this was carried out per principle until 2020 and also the concomitant pancreaticoduodenectomy in 6.2% (13/209) of the patients. An R1 resection was confirmed in 19.3% (40/209) of the overall cohort. Major complications (Clavien-Dindo ≥ IIIb) after surgery were observed in 40.2% (84/209) of the patients. Perioperative mortality was observed in 32 out of 209 patients (15.3%). Further demographic and clinicopathological details of the cohort are outlined in Table 1.

**Table 1:** Patients’ characteristics.

Demographics	Overall cohort (n = 209)	Long-term survival subgroup (n = 27)	Poor outcome subgroup (n = 55)
Gender, m/f (%)	139 (66.5) / 70 (33.5)	18 (66.7) / 9 (33.3)	35 (63.6) / 20 (36.4)
Age (years)	68 (58–74)	69 (59–75)	68 (57–73)
BMI (kg/m^2^)	25 (23–28)	24 (22–26)	26 (24–29)
Bismuth classification, n (%)			
I	12 (5.8)	1 (3.7)	4 (7.3)
II	28 (13.4)	1 (3.7)	8 (14.5)
IIIa	61 (29.2)	10 (37.0)	14 (25.5)
IIIb	51 (24.4)	8 (29.6)	14 (25.5)
IV	57 (27.3)	7 (25.9)	15 (27.3)
Neoadjuvant therapy, n (%)	10 (4.8)	1 (3.7)	1 (1.8)
Portal vein embolization, n (%)	74 (35.4)	12 (44.4)	19 (34.5)
ASA, n (%)			
I	8 (3.8)	2 (7.4)	1 (1.9)
II	71 (34.0)	11 (40.7)	18 (33.3)
III	116 (55.5)	14 (51.9)	29 (53.7)
IV	14 (6.7)	0	6 (11.1)
V	0	0	
Preoperative cholangitis, n (%)	67 (32.1)	4 (14.8)	18 (32.7)
Preoperative EBD, n (%)	150 (71.8)	17 (63.0)	41 (74.5)
Preoperative PBD, n (%)	52 (24.9)	7 (25.9)	12 (21.8)
Portal vein infiltration > 180°, n (%)			
None	117 (56.3)	21 (77.8)	25 (46.3)
Main	3 (1.4)	0	1 (1.9)
Bifurcation	27 (13.0)	1 (3.7)	5 (9.3)
Right	22 (10.6)	3 (11.1)	9 (16.7)
Left	38 (18.3)	2 (7.4)	14 (25.9)
Right and left	1 (0.5)	0	
Arterial infiltration > 180°, n (%)			
None	169 (81.3)	23 (85.2)	42 (77.8)
Main	0	0	0
Bifurcation	0	0	0
Right	34 (16.3)	3 (11.1)	11 (20.4)
Left	3 (1.4)	0	1 (1.9)
Right and left	2 (1.0)	1 (3.7)	0
Lobar atrophy, n (%)			
None	146 (70.2)	18 (66.7)	40 (74,1)
Right	9 (4.3)	1 (3.7)	2 (3.7)
Left	53 (25.5)	8 (29.6)	12 (22.2)
sFLR (%)	57 (40–72)	48 (36–62)	59 (39–79)
**Clinical chemistry**			
Albumin (g/dl)	38 (34–41)	40 (36–42)	38 (33–41)
AST (U/l)	45 (34–84)	47 (31–90)	50 (37–95)
ALT (U/l)	58 (35–110)	81 (44–148)	65 (44–134)
GGT (U/l)	403 (188–758)	434 (174–771)	472 (248–771)
Total bilirubin (mg/dl)	1.1 (0.6–2.8)	0.9 (0.6–1.7)	1.1 (0.5–2.9)
Platelet count (/nl)	295 (228–389)	276 (212–378)	302 (232–388)
Alkaline Phosphatase (U/l)	266 (158–423)	267 (178–389)	251 (180–412)
Prothrombin time (%)	96 (84–105)	99 (86–112)	94 (82–105)
INR	1.03 (0.97–1.11)	1.01 (0.92–1.10)	1.04 (0.97–1.14)
Hemoglobin (g/dl)	12.2 (11.0–13.3)	13.0 (12.1–13.6)	11.6 (10.2–13.1)
CRP (mg/l)	12 (6–36)	11 (5–34)	10 (6–29)
**Operative Data**			
Operative time (minutes)	450 (379–511)	375 (327–450)	456 (390–510)
Operative procedure, n (%)			
Limited bile duct resection	8 (3.8)	0	3 (5.5)
Right hepatectomy	26 (12.4)	2 (7.4)	7 (12.7)
Left hepatectomy	28 (13.4)	3 (11.1)	9 (16.4)
Mesohepatectomy	2 (1.0)		
Extended right hepatectomy	42 (20.1)	7 (25.9)	9 (16.4)
Extended left hepatectomy	53 (25.4)	9 (33.3)	10 (18.2)
Right trisectionectomy	26 (12.4)	4 (14.8)	8 (14.5)
Left trisectionectomy	9 (4.3)	0	5 (9.1)
Hepatoduodenectomy	13 (6.2)	2 (7.4)	4 (7.3)
ALPPS	2 (1.0)	0	0
Portal vein reconstruction	169 (80.9)	27 (100)	47 (85.5)
Arterial reconstruction	16 (7.7)	2 (7.4)	5 (9.1)
Intraoperative PRBC	0 (0–2)	0 (0–0)	2 (0–2)
Intraoperative FFP	2 (0–4)	0 (0–3)	3 (0–4)
**Pathological examination**			
R1 resection, n (%)	40 (19.3)	1 (3.7)	16 (29.1)
pT category, n (%)			
1	17 (8.2)	4 (14.8)	1 (1.8)
2	120 (58.0)	16 (59.3)	29 (52.7)
3	51 (24.6)	6 (22.2)	16 (29.1)
4	19 (9.2)	1 (3.7)	9 (16.4)
Tumor size (mm)	30 (20–45)	26 (16–41)	34 (25–45)
pN category			
N0	116 (55.8)	22 (81.5)	25 (45.5)
N1	92 (44.2)	5 (18.5)	30 (54.5)
Tumor grading, n (%)			
G1	9 (4.5)	2 (7.7)	1 (1.9)
G2	138 (68.7)	22 (84.6)	36 (67.7)
G3	53 (26.3)	2 (7.7)	15 (29.6)
G4	1 (0.5)	0	1 (1.9)
MVI, n (%)	62 (30.4)	18 (81.8)	26 (48.1)
LVI, n (%)	46 (23.0)	2 (7.4)	21 (40.4)
PNI, n (%)	1 (81.2)	18 (81.8)	41 (91.1)
**Postoperative Data**			
Intensive care, days	2 (1–5)	1 (1–2)	2 (1–4)
Hospitalization, days	18 (12–34)	15 (12–23)	26 (14–37)
Postoperative complications, n (%)			
No complications	35 (16.7)	8 (29.6)	4 (7.3)
Clavien-Dindo I	12 (5.7)	2 (7.4)	3 (5.5)
Clavien-Dindo II	42 (20.1)	7 (25.9)	15 (27.3)
Clavien-Dindo IIIa	36 (17.2)	5 (18.5)	14 (25.5)
Clavien-Dindo IIIb	34 (16.3)	3 (11.1)	13 (23.6)
Clavien-Dindo IVa	11 (5.3)	2 (7.4)	3 (5.5)
Clavien-Dindo IVb	7 (3.3)	0	3 (5.5)
Clavien-Dindo V	32 (15.3)	0	0
**Oncologic Data** *			
Adjuvant therapy	57 (27.3)	1 (3.7)	18 (32.7)
Median RFS, months (95% CI)	36 (22–50)	123* (112–135)	10 (8–12)
Median CSS, months (95% CI)	32 (20–44)	125* (113–136)	16 (12–20)
CA19-9 (kU/l)	98.5 (35–335)	67 (12–135)	184 (52–537)

Data presented as median and interquartile range if not noted otherwise. Transfusion data refers to intraoperative application. ALT, alanine aminotransferase; ASA, American society of anesthesiologists classification; AST, aspartate aminotransferase; BMI, body mass index;; CSS, cancer-specific survival; OS, overall survival; EBD, endoscopic biliary drainage; FFP, fresh frozen plasma; pCCA, perihilar cholangiocarcinoma; GGT, gamma glutamyltransferase; INR, international normalized ratio; LVI, lympho-vascular invasion; MVI, microvascular invasion; PBD, percutaneous biliary drainage; PNI, perineural invasion; RFS, recurrence-free survival.

*mean

### Long-term survival and poor oncological outcome subgroups

Patients with perioperative mortality were excluded from the cohort (32/209). 12.9% of the whole cohort were defined as LTS (27/209). 26.3% of the whole cohort showed poor oncological outcome (55/209). Patients not included in these subgroups died perioperatively (n = 32, 15.3%), had missing recurrence information (n = 4, 1.9%), showed intermediate oncological outcome (n = 24, 11.5%), died of a not cancer-related cause within the first 5 years of follow-up (n = 24, 11.5%) or showed no signs of tumor recurrence, but follow up was less than 5 years (n = 43, 20.6%). Demographic and clinicopathological details of the two subgroups are shown in Table 1.

### Comparative logistic regression and definition of the prognostic composite variable

To determine key differences between patients with long-term survival and poor oncological outcome, logistic regressions were carried out. Here, prognostic variables for poor oncological outcome were portal vein infiltration (HR = 4.06, p = 0.009), low preoperative hemoglobin (HR = 0.19, p = 0.002), long operative time (HR = 4.64, p = 0.003), intraoperative transfusion of fresh frozen plasma (FFP, HR = 3.8, p = 0.007) and packed red blood cells (PRBC, HR = 5.67, p = 0.001), R1 resection (HR = 10.66, p = 0.026), larger tumors (HR = 3.96, p = 0.012), positive nodal status (HR = 5.28, p = 0.003), dedifferentiated tumors (HR = 5.83, p = 0.026), microvascular invasion (MVI, HR = 4.24, p = 0.011), lymphovascular invasion (HR = 8.47, p = 0.007), prolonged intensive care unit (ICU) time (HR = 3.95, p = 0.015), prolonged hospitalization (HR = 4.29, p = 0.005) and adjuvant therapy (HR = 12.65, p = 0.017). These variables were included in a multivariable analysis where portal vein infiltration (HR = 5.85, p = 0.018) and PRBC transfusions (HR = 10.29, p = 0.002) were identified as independently prognostic variables. More details are presented in Table 2.

**Table 2:** Logistic regression for long-term survival vs. short-term cancer specific death.

	Univariate analysis		Multivariate analysis	
	HR (95% CI)	*P* value	HR (95% CI)	*P* value
**Demographics**				
Sex (male = 1)	1.14 (0.34–3.02)	.787		
Age (≤ 65 years = 1)	0.93 (0.36–2.37)	.874		
BMI (≤ 25 kg/m^2^ = 1)	2.55 (0.99–6.59)	.053		
Bismuth type (I/II = 1)	1.07 (0.38–3.05)	.897		
Neoadjuvant therapy (no = 1)	0.48 (0.03–8.01)	.610		
PVE (no = 1)	1.52 (0.59–3.88)	.386		
ASA (I/II = 1)	1.63 (0.64–4.13)	.308		
Preoperative cholangitis (no = 1)	0.36 (0.11–1.19)	.093		
EBD (no = 1)	0.58 (0.22–1.56)	.281		
PBD (no = 1)	1.25 (0.43–3.67)	.679		
Portal vein infiltration > 180° (no = 1)	4.06 (1.42–11.64)	**.009**	5.85 (1.35–25.32)	**.018**
Arterial infiltration > 180° (no = 1)	1.64 (0.48–5.68)	.433		
Lobar atrophy (no = 1)	0.70 (0.26–1.91)	.487		
sFLR (≤ 40% = 1)	0.84 (0.31–2.27)	.734		
**Clinical chemistry**				
Albumin (≤ 35 g/l = 1)	0.40 (0.13–1.24).	.112		
AST (≤ 50 U/l = 1)	1.40 (0.55–3.56)	.477		
ALT (≤ 50 U/l = 1)	0.54 (0.15–1.97)	.352		
GGT (≤ 400 U/l = 1)	1.30 (0.51–3.43)	.580		
Bilirubin (≤ 1 mg/dl = 1)	1.62 (0.64–4.12)	.309		
Alkaline phosphatase (≤ 250 U/l = 1)	0.89 (0.34–2.28)	.890		
Platelet count (≤ 300 /nl = 1)	1.62 (0.64–4.12)	.309		
INR (≤ 1 = 1)	2.09 (0.80–5.49)	.135		
Hemoglobin (≤ 12 g/dl = 1)	0.19 (0.07–0.55)	**.002**		.463
CRP, mg/l (≤ 10 mg/l = 1)	0.96 (0.38–2.46)	.937		
**Operative data**				
Operative time (≤ 360 min = 1)	4.63 (1.67–12.81)	**.003**		.073
Type of resection		.140		
Right/Left hepatectomy	1			
Extended hepatectomy	0.43 (0.14–1.32))			
Hepatoduodenectomy	1.25 (0.23–6.90)	.798		
Vascular resection (no = 1)	1.25 (0.23–6.90)	.798		
FFP transfusion (no = 1)	3.80 (1.44–10.01)	**.007**		.077
PRBC transfusion (no = 1)	5.67 (1.97–16.32)	**.001**	10.29 (2.34–45.16)	.**002**
**Pathological data**				
R1 resection (no = 1)	10.66 (1.33–85.41)	**.026**		.398
pT category (T1/T2 = 1)	2.26 (0.82–6.25)	.115		
Tumorsize (≤ 30 mm = 1)	3.96 (1.35–11.61)	**.012**		.378
pN category (N0 = 1)	5.28 (1.75–15.97)	**.003**		.273
Tumor grading (G1/G2 = 1)	5.83 (1.23–27.59)	**.026**		.565
MVI (no = 1)	4.24 (1.40–12.86)	**.011**		.227
LVI (no = 1)	8.47 (1.81–39.62)	**.007**		.998
PNI (no = 1)	2.28 (0.51–10.133)	.280		
**Postoperative data**				
ICU time (≤ 1 days = 1)	3.95 (1.30–11.92)	**.015**		.075
Hospitalization (≤ 21 days = 1)	4.29 (1.55–11.83)	**.005**		.073
Complications (≤ 3a = 1)	2.32 (0.76–7.11)	.140		
**Oncological data**				
Adjuvant therapy (no = 1)	12.65 (1.59–100.7)	.**017**		.065
CA19-9 (> 263 kU/l = 1)	5.79 (0.65–51.51)	.115		

Various parameters are associated with cancer specific survival. Transfusion data refers to intraoperative application. ALT, alanine aminotransferase; ASA, American society of anesthesiologists classification; AST, aspartate aminotransferase; BMI, body mass index; CRP, c-reactive protein; EBD, endoscopic biliary drainage; FFP, fresh frozen plasma; GGT, gamma glutamyltransferase; ICU, intensive care unit; INR, international normalized ratio; LVI, lympho-vascular invasion; OS, overall survival; MVI, microvascular invasion; PBD, percutaneous biliary drainage; PNI, perineural invasion; PVE, portal vein embolization

Based on these results, a composite variable for poor oncological outcome (portal vein infiltration AND intraoperative PRBC transfusion) was defined and used for further analysis. Of note, the composite variable is based on radiologically assessed PVI, while pathologically confirmed PVI was present in 33.8% (70/209) patients of the whole cohort, 25.9% (7/27) individuals of the LTS group and 45.5% (25/55) patients of the STS group. Factors associated with PRBC are analyzed in S1 Table.

### Univariate and multivariable logistic regressions of the overall cohort

To validate the prognostic value of the defined composite variable, the univariate and multivariable Cox regression analyses were conducted in the overall cohort. For CSS, high ASA score (HR = 1.51, p = 0.047), preoperative cholangitis (HR = 0.58, p = 0.005), portal vein infiltration (HR = 2.02, p<0.001, arterial infiltration (HR = 1.60, p = 0.038), low albumin levels (HR = 0.64, p = 0.34), elevated INR (HR = 1.81, p = 0.006), low hemoglobin levels (HR = 0.65, p = 0.028), long operative time (HR = 1.63, p = 0.018), intraoperative FFP (HR = 2.57, p<0.001) as well as PRBC (HR = 2.44, p<0.001) transfusion, R1 resection (HR = 2.01, p = 0.002), higher pT category (HR = 2.24, p<0.001), tumor size ≤ 30 mm (HR = 2.42, p<0.001), pN category (HR = 1.89, p<0.001), tumor grading (HR = 2.13, p<0.001), microvascular invasion (MVI, HR = 2.18, p<0.001), lymphovascular invasion (LVI, HR = 1.77, p = 0.011), ICU time (HR = 2.22, p<0.001), duration of hospitalization (HR = 1.77, p = 0.005), perioperative complications (HR = 3.05, p<0.001) as well as the composite variable (HR = 0.28, p<0.001) were associated with decreased CSS (Table 3). All variables showing a statistical significance were further transferred in a multivariable Cox regression model. Here, pN category (HR = 2.15, p<0.001), postoperative complications (HR = 3.06, p<0.001) and the prognostic composite variable (HR = 0.35, p<0.001) were independent predictors of CSS.

**Table 3:** Univariate and multivariate analysis of cancer-specific survival.

	Univariate analysis		Multivariate analysis	
	HR (95% CI)	*P* value	HR (95% CI)	*P* value
**Demographics**				
Sex (male = 1)	0.97 (0.65–1.46)	.893		
Age (≤ 65 years = 1)	1.07 (0.73–1.57)	.725		
BMI (≤ 25 kg/m^2^ = 1)	1.16 (0.79–1.69)	.447		
Bismuth type (I/II = 1)	1.20 (0.80–1.80)	.367		
Neoadjuvant therapy (no = 1)	0.60 (0.19–1.90)	.382		
PVE (no = 1)	0.96 (0.65–1.42)	.825		
ASA (I/II = 1)	1.51 (1.01–2.26)	.**047**		.153
Preoperative cholangitis (no = 1)	0.58 (0.39–0.85)	.**005**		.166
EBD (no = 1)	0.79 (0.50–1.23)	.283		
PBD (no = 1)	0.81 (0.53–1.23)	.322		
Portal vein infiltration > 180° (no = 1)	2.02 (1.38–2.96)	**< .001**		.253
Arterial infiltration > 180° (no = 1)	1.60 (1.03–2.49)	**.038**		.454
Lobar atrophy (no = 1)	1.00 (0.66–1.52)	.996		
sFLR (≤ 40% = 1)	0.92 (0.59–1.42)	.705		
**Clinical chemistry**				
Albumin (≤ 35 g/l = 1)	0.64 (0.43–0.97)	**.034**		.490
AST (≤ 50 U/l = 1)	0.90 (0.61–1.33)	.607		
ALT (≤ 50 U/l = 1)	0.75 (0.49–1.15)	.188		
GGT (≤ 400 U/l = 1)	0.98 (0.66–1.45)	.979		
Bilirubin (≤ 1 mg/dl = 1)	1.36 (0.93–1.99)	.116		
Alkaline phosphatase (≤ 250 U/l = 1)	1.05 (0.71–1.55)	.826		
Platelet count (≤ 300 /nl = 1)	0.97 (0.66–1.42)	.966		
INR (≤ 1 = 1)	1.81 (1.19–2.76)	**.006**		.070
Hemoglobin (≤ 12 g/dl = 1)	0.65 (0.44–0.96)	.**028**		.431
CRP, mg/l (≤ 10 mg/l = 1)	1.37 (0.92–2.03)	.120		
**Operative data**				
Operative time (≤ 360 min = 1)	1.63 (1.09–2.43)	**.018**		.071
Type of resection				
Right/Left hepatectomy	1			
Extended hepatectomy	0.99 (0.66–1.52)	.997		
Hepatoduodenectomy	1.43 (0.72–2.84)	.302		
Vascular resection (no = 1)	1.74 (0.95–3.17)	.071		
FFP transfusion (no = 1)	2.57 (1.70–3.89)	**< .001**		.074
PRBC transfusion (no = 1)	2.44 (1.65–3.62)	**< .001**		.525
**Pathological data**				
R1 resection (no = 1)	2.01 (1.28–3.15)	**.002**		.344
pT category (T1/T2 = 1)	2.24 (1.53–3.29)	**< .001**		.137
Tumorsize (≤ 30 mm = 1)	2.42 (1.62–3.62)	**< .001**		.320
pN category (N0 = 1)	1.89 (1.29–2.77(	**.001**	2.15 (1.36–3.41)	**.001**
Tumor grading (G1/G2 = 1)	2.13 (1.39–3.26)	**< .001**		.091
MVI (no = 1)	2.18 (1.47–3.25)	**< .001**		.177
LVI (no = 1)	1.77 (1.14–2.74)	**.011**		.130
PNI (no = 1)	1.77 (0.99–3.19)	.056		
**Postoperative data**				
ICU time (≤ 1 days = 1)	2.22 (1.51–3.27)	**< .001**		.068
Hospitalization (≤ 21 days = 1)	1.77 (1.91–2.63)	**.005**		.455
Complications (≤ 3a = 1)	3.05 (2.08–4.49)	**< .001**	3.06 (1.91–4.89)	**< .001**
**Oncological data**				
Adjuvant therapy (no = 1)	0.63 (0.39–1.02)	.058		
CA19-9 (> 263 kU/l = 1)	1.49 (0.86–2.60)	.158		excl.
**Prognostic composite variable**				
Predictor	0.28 (0.19–0.43)	**< .001**	0.35 (0.21–0.57)	**< .001**

Various parameters are associated with cancer specific survival. Transfusion data refers to intraoperative application. CA19-9 was excluded from the multivariate analysis as it was only available for 60% of the cases. ALT, alanine aminotransferase; ASA, American society of anesthesiologists classification; AST, aspartate aminotransferase; BMI, body mass index; CRP, c-reactive protein; EBD, endoscopic biliary drainage; FFP, fresh frozen plasma; GGT, gamma glutamyltransferase; ICU, intensive care unit; INR, international normalized ratio; LVI, lympho-vascular invasion; OS, overall survival; MVI, microvascular invasion; PBD, percutaneous biliary drainage; PNI, perineural invasion; PVE, portal vein embolization

A similar approach was conducted for RFS. In the univariate analysis, PV infiltration (HR = 1.61, p = 0.034), albumin levels (HR = 0.59, p = 0.026), INR (HR = 1.61, p = 0.046), hemoglobin (HR = 0.43, p<0.001), both FFP (HR = 2.26, p<0.001) and PRBC (HR = 2.18, p<0.001) transfusion, R1 resection (HR = 2.43, p<0.001), pT category (HR = 2.17, p<0.001), tumorsize (HR = 2.02, p = 0.002), pN category (HR = 2.35, p<0.001), tumor grading (HR = 2.60, p<0.001), MVI (HR = 2.69, p<0.001), LVI (HR = 2.53, p<0.001), perineural invasion (PNI, HR = 2.11, p = 0.037), hospitalization (HR = 2.07, p = 0.001), CA 19–9 (HR = 2.14, p = 0.014) and the prognostic composite variable (HR = 0.30, p<0.001) showed a statistical significance (Table 4). In multivariate analysis, hemoglobin (HR = 0.52, p = 0.018), tumor grading (HR = 2.09, p = 0.013), LVI (HR = 3.71, p<0.001), hospitalization (HR = 2.35, p = 0.003) and the prognostic composite variable (HR = 0.27, p<0.001) were identified to be independent predictors for RFS (Table 4).

**Table 4:** Univariate and multivariate analysis of recurrence-free survival.

	Univariate analysis		Multivariate analysis	
	HR (95% CI)	*P* value	HR (95% CI)	*P* value
**Demographics**				
Sex (male = 1)	1.02 (0.65–1.61)	.930		
Age (≤ 65 years = 1)	0.88 (0.57–1.36)	.572		
BMI (≤ 25 kg/m^2^ = 1)	1.46 (0.94–2.26)	.094		
Bismuth type (I/II = 1)	1.04 (0.65–1.67)	.872		
Neoadjuvant therapy (no = 1)	0.48 (0.12–1.96)	.306		
PVE (no = 1)	1.00 (0.63–1.57)	.982		
ASA (I/II = 1)	1.17 (0.75–1.82)	.500		
Preoperative cholangitis (no = 1)	0.66 (0.42–1.04)	.073		
EBD (no = 1)	0.94 (0.58–1.53)	.800		
PBD (no = 1)	0.93 (0.55–1.55)	.770		
Portal vein infiltration > 180° (no = 1)	1.61 (1.04–2.50)	**.034**		.746
Arterial infiltration > 180° (no = 1)	1.42 (0.83–2.43)	.197		
Lobar atrophy (no = 1)	0.87 (0.53–1.43)	.577		
sFLR (≤ 40% = 1)	0.93 (0.57–1.52)	.766		
**Clinical chemistry**				
Albumin (≤ 35 g/l = 1)	0.59 (0.37–0.94)	**.026**		.270
AST (≤ 50 U/l = 1)	1.38 (0.89–2.12)	.150		
ALT (≤ 50 U/l = 1)	0.99 (0.59–1.64)	.963		
GGT (≤ 400 U/l = 1)	0.99 (0.64–1.56)	.980		
Bilirubin (≤ 1 mg/dl = 1)	1.42 (0.92–2.19)	.118		
Alkaline phosphatase (≤ 250 U/l = 1)	1.07 (0.68–1.67)	.780		
Platelet count (≤ 300 /nl = 1)	1.37 (0.89–2.12)	.156		
INR (≤ 1 = 1)	1.61 (1.01–2.56)	**.046**		.170
Hemoglobin (≤ 12 g/dl = 1)	0.43 (0.28–0.67)	**< .001**	0.52 (0.30–0.89)	**.018**
CRP, mg/l (≤ 10 mg/l = 1)	1.03 (0.66–1.60)	.911		
**Operative data**				
Operative time (≤ 360 min = 1)	1.47 (0.94–2.31)	.091		
Type of resection		.991		
Right/Left hepatectomy	1			
Extended hepatectomy	1.00 (0.62–1.61)			
Hepatoduodenectomy	1.63 (0.75–3.54)	.218		
Vascular resection (no = 1)	1.40 (0.65–3.04)	.395		
FFP transfusion (no = 1)	2.26 (1.43–3.57)	**< .001**		.090
PRBC transfusion (no = 1)	2.18 (1.40–3.38)	**< .001**		.738
**Pathological data**				
R1 resection (no = 1)	2.43 (1.47–4.04)	**< .001**		.778
pT category (T1/T2 = 1)	2.17 (1.40–3.37)	**< .001**		.824
Tumorsize (≤ 30 mm = 1)	2.02 (1.28–3.20)	**.002**		.418
pN category (N0 = 1)	2.35 (1.51–3.64)	**< .001**		.080
Tumor grading (G1/G2 = 1)	2.60 (1.60–4.21)	**< .001**	2.09 (1.17–3.75)	**.013**
MVI (no = 1)	2.69 (1.71–4.22)	**< .001**		.372
LVI (no = 1)	2.53 (1.56–4.09)	**< .001**	3.71 (2.04–6.73)	**< .001**
PNI (no = 1)	2.11 (1.05–4.27)	**.037**		.188
**Postoperative data**				
ICU time (≤ 1 days = 1)	1.54 (0.98–2.40)	.060		
Hospitalization (≤ 21 days = 1)	2.07 (1.33–3.24)	**.001**	2.35 (1.35–4.09)	**.003**
Complications (≤ 3a = 1)	1.40 (0.87–2.24)	.167		
**Oncological data**				
Adjuvant therapy (no = 1)	1.40 (0.88–2.23)	.154		
CA19-9 (> 263 kU/l = 1)	2.14 (1.17–3.91)	**.014**		excl.
**Prognostic composite variable**				
Predictor	0.30 (0.18–0.49)	**< .001**	0.27 (0.15–0.49)	**< .001**

Various parameters are associated with recurrence-free survival. Transfusion data refers to intraoperative application. CA19-9 was excluded from the multivariate analysis as it was only available for 60% of the cases. ALT, alanine aminotransferase; ASA, American society of anesthesiologists classification; AST, aspartate aminotransferase; BMI, body mass index; CRP, c-reactive protein;; EBD, endoscopic biliary drainage; FFP, fresh frozen plasma; GGT, gamma glutamyltransferase; ICU, intensive care unit; INR, international normalized ratio; LVI, lympho-vascular invasion; OS, overall survival; MVI, microvascular invasion; PBD, percutaneous biliary drainage; PNI, perineural invasion; PVE, portal vein embolization

### Survival analysis

**Fig 1:**
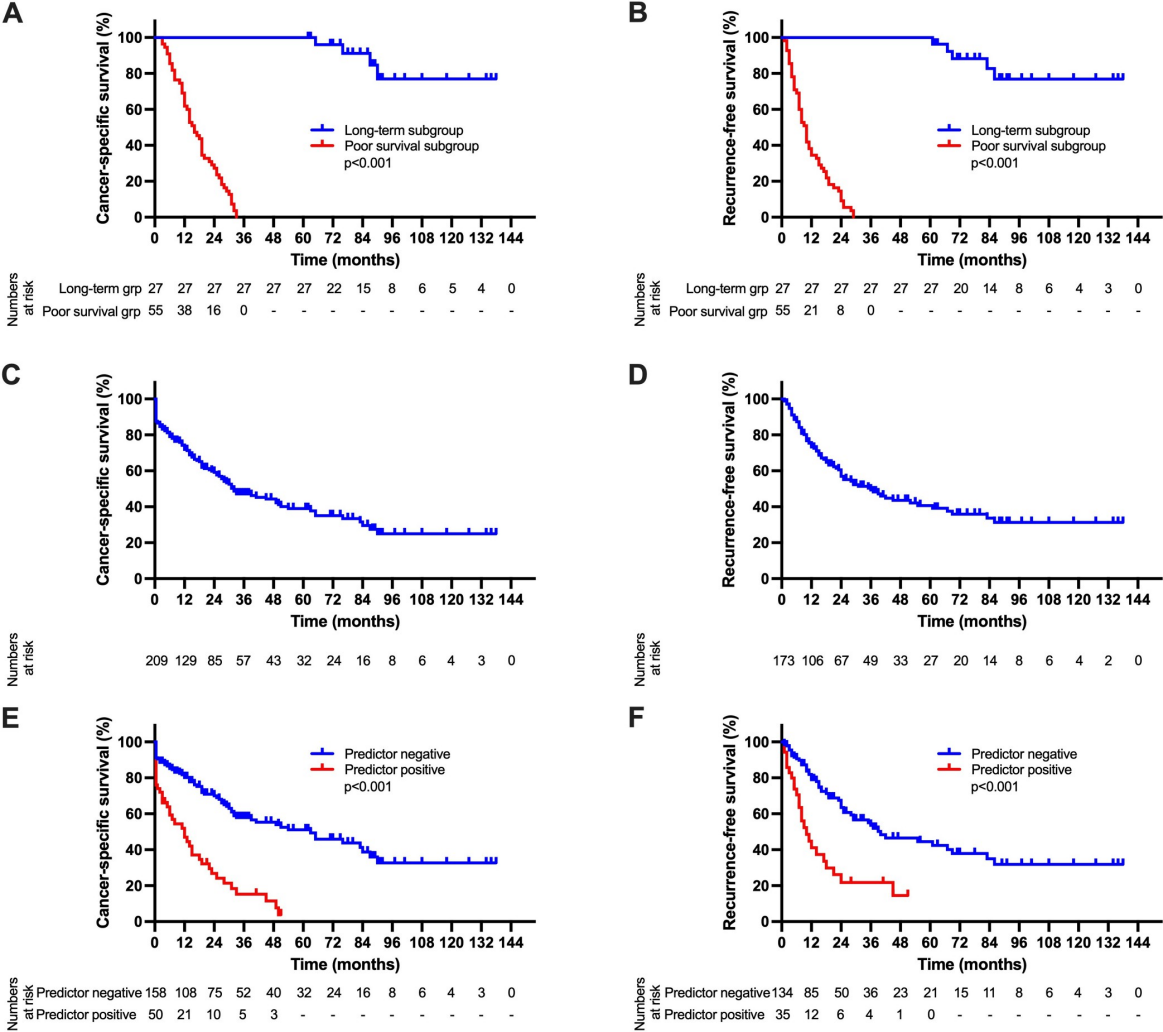
Oncological survival in perihilar cholangiocarcinoma. **A: Cancer-specific survival in long-term vs. short-term survivors**. The mean CSS in the LTS group was 125 months and the median CSS was 16 months in the STS group (p<0.001). **B: Recurrence-free survival in long-term vs. short-term survivors**. The mean RFS in the LTS group was 123 months and the median RFS was 125 months in the STS group (p<0.001). **C: Cancer-specific survival in the overall cohort**. The median CSS was 32 months in the overall cohort. **D: Recurrence-free survival in the overall cohort**. The median RFS was 36 months (perioperatively deceased patients were excluded from the RFS analysis). **E: Cancer-specific survival stratified by the prognostic composite variable**. The median CSS was 12 months in predictor positive patients and 63 months in predictor negative patients (p<0.001) **F: Recurrence-free survival stratified by the prognostic composite variable**. The median RFS was 10 months in predictor positive patients and 40 months in predictor negative patients (p<0.001). The prognostic composite variable is defined positive in case of intraoperative PRBC transfusions and preoperative portal vein invasion and negative in all other cases. CI, confidence interval; CSS, cancer-specific survival; pCCA, perihilar cholangiocarcinoma; RFS, recurrence-free survival.

Survival analysis was conducted to illustrate the prognostic value of the defined composite variable. In a comparative analysis between LTS and STS group, the mean CSS was 125 (CI: 114–136) months in the LTS group compared to a median CSS of 16 (CI: 12–20) months in the STS group (p<0.001, Fig 1A). Furthermore, the mean RFS in the LTS group was 123 (CI: 112–135) months and the median RFS 10 (CI: 8–12) months in the STS group (p<0.001, Fig 1B). In the overall cohort, the median CSS was 32 (CI: 20–44) months (Fig 1C). The median RFS was 36 (CI: 22–50) months (Fig 1D). In an analysis regarding the defined prognostic composite variable, the median CSS was 12 (CI: 5–19) months in predictor positive patients and 63 (CI: 33–93) months in predictor negative patients (p<0.001, Fig 1E). The median RFS was 10 (CI: 5–15) months in predictor positive patients and 40 (CI: 5–15) months in predictor negative patients (p<0.001, Fig 1F).

## Discussion and conclusion

PCCA accounts for the majority of biliary tract cancers with an incidence of up to 70% [25]. The current gold standard therapy remains the radical surgical resection for localized tumors which provides favorable oncological outcomes in comparison to other treatment modalities such as systemic chemotherapy [26]. Due to the significant improvements of not only surgical techniques but also interventional management over the past decades, the number of patients who undergo surgery with more advanced tumors has remarkably increased [27]. However, this might also come at a price of higher perioperative morbidity and mortality as arterial and or (portal)venous resection and reconstruction are commonly used to treat locally progressed tumors [26, 28–30]. This development gives rise for concerns regarding oncological prognosis and long-term outcome after extensive surgery for pCCA. Thus, we investigated predictors of oncological outcome with respect to long-term survival in a large European monocentric cohort of 209 patients who underwent surgical treatment for pCCA.

Here, we identified a subgroup of 12.9% (n = 27; also due to limited follow-up in the cohort) of the whole cohort as LTS with a mean RFS of 123 months and mean CSS of 125 months and identified preoperative portal vein infiltration (OR = 5.85, p = 0.018) and intraoperative transfusion of PRBC (OR = 10.29, p = 0.002) as the most distinct feature differentiating this group from a poor outcome sub cohort. In the overall patient set, the combination of both variables (combined as prognostic composite variable) showed excellent prognostic ability and was able to define patients who did not experience long-term survival in our cohort. Moreover, in univariate and multivariate analyses pN category (HR = 2.15, p<0.001), postoperative complications (HR = 3.06, p<0.001) and the prognostic composite variable (HR = 0.35, p<0.001) appear to be independent predictors of CSS. For RFS, hemoglobin (HR = 0.52, p = 0.018), tumor grading (HR = 2.09, p = 0.013), LVI (HR = 3.71, p<0.001), hospitalization (HR = 2.35, p = 0.003) and the prognostic composite variable (HR = 0.27, p<0.001) were identified to be independent predictors for RFS.

Due to the close anatomical relationship between the biliary tree and major vessels (hepatic artery and PV) in the liver hilum, PV involvement in patients with pCCA is not uncommon. The detection rate of PV involvement through radiographic imaging in patients with pCCA has a sensitivity of up to 89% and specificity of 92% [31–33]. However, radiologic assessment of PVI highly differs between radiologists [33–35]. Adequately identifying PVI preoperatively is crucial for the surgical planning process since multiple factors are to be considered in such case (e.g. resectability, risk of portal vein thrombosis, possible impaired liver function due to reduced hepatic blood flow). There is controversial data available regarding the influence of PVI on oncological outcome [28, 36, 37]. Van Vught et al. reported a large cohort of 674 patients with pCCA of which more than half of the patients displayed PVI [7]. PVI was shown to have a significant effect on OS in Kaplan-Meier and univariate Cox regression analysis. However, in the subset of patients undergoing surgical resection (n = 155), PVI was not shown to have an adverse effect on both short- and long-term outcomes. In contrast, our group has reported a negative effect of portal vein thrombosis as the “end-stage” of PVI on CSS, RFS and perioperative mortality [38]. It has to be noted that there is a difference between radiological and pathological portal vein filtration. Preoperative assessment of PVI typically involves radiological evaluation, often using CT imaging. In a study by Wattanasateriri et al., preoperative radiological assessment of PVI was compared to postoperative pathological confirmation in 62 patients undergoing surgery for pCCA. Their analysis revealed that out of 18 cases assessed as negative for PVI preoperatively, 4 cases were false negatives (i.e., underestimated). Additionally, 5 out of 25 cases assessed as positive for PVI preoperatively were false positives (i.e., overestimated), as no involvement was confirmed histologically [39]. In another study conducted by Masselli et al., MR radiological assessment was compared to pathological analysis of PVI in pCCA patients undergoing surgery. Out of 15 cases, PVI was underestimated on MRI in 3 cases. Additionally, in one case, PVI was overestimated and reported as infiltrated on MRI, although histological examination did not confirm this finding [40]. There are several reasons for the discrepancy between radiological and pathological PVI findings. Radiological imaging might be limited due to factors such as image resolution, artifacts and interpretation by different radiologists. Moreover, radiological imaging provides solely a snapshot of the tumor at a specific point of time. When surgery is carried out later, tumor growth might have taken place. From a histopathological point of view, the investigated tissue sample might not adequately represent the entire extent of tumor involvement, leading to false-negative results through sampling bias. Another reason might depict the complex anatomy of the liver hilum having overlapping structures (bile ducts, vessels) that can pose difficulties in distinguishing between the structures. Also, effects of cholangitis, peritumoral fibrosis near the portal vein or largely dilatated bile ducts compressing the portal vein might interfere with the evaluation.

While the presence of PVI is less investigated, the role of portal vein resection in pCCA patients was more in the center of interest. Here, conflicting results have been reported as well. In a recent meta-analysis of 17 studies, portal vein resection itself was associated with impaired oncological outcome. However, if controlled for heterogeneity and focused on recent studies, no impact on oncological outcome was observed [41]. Given the heterogeneity in definition of PVI, differences in both pre- and perioperative management and conflicting long-term results, no final conclusion can be drawn regarding the oncological impact of PVI in pCCA patients. However, given our observation that PVI might be of importance regarding long-term outcome, it is advocated to regularly assess and take PVI into consideration for perioperative decision-making.

The impact of allogenic blood transfusion in pCCA patients is equally controversial. Blood transfusions are known to have immunosuppressive effects, which can result in tumor recurrence and dismal long-term oncological outcomes [42, 43]. For example, Liu et al. investigated the impact of intraoperative PRBC transfusion or PRBC transfusion within 7 days after surgery in pCCA patients on long-term survival (>5-year OS) in a multicenter design and demonstrated a negative impact in early stage pCCA (AJCC stage I) [44]. In contrast, Dekker et al. were not able to show a negative effect on OS and disease recurrence [45]. Similarly, no effect on oncological outcome has been observed in a German series comprising pCCA, intrahepatic CCA (iCCA) and ductal CCA (dCCA) patients as well as in a Chinese data set investigating iCCA [46, 47]. In a recent analysis of our group investigating iCCA and pCCA, we were able to demonstrate a negative effect of in-hospital allogenic blood transfusions on CSS which also increased with quantitively more transfusions during hospitalization [16]. However, this cohort was based on two subtypes of CCA and fresh frozen plasma showed a more pronounced effect on oncological outcome than blood transfusions. However, these results must be strictly separated from this analysis as only intraoperative transfusions were assessed here while the above-mentioned study analyzed the whole duration of hospitalization (intra- and postoperatively) quantitatively and qualitatively [16]. As for PVI, the currently available data do not allow a definite conclusion about the impact of allogenic blood transfusions on oncological outcome, as multiple definitions (intraoperative, perioperative, in-hospital) were historically applied and different transfusions strategies might interfere with the interpretation of the data.

As illustrated above, there is currently no final verdict on the impact of PRBC on oncological outcome in the literature as conflicting reports are available with our data indicating a notable prognostic effect. A frequent argument is that the requirement of PRBC rather reflects clinical circumstances which also impacts outcomes than having an intrinsic adverse effect on its own [45]. This idea is supported by one large study of veterans undergoing surgery showing no effect of PRBC on OS when the analysis was adjusted for perioperative complications [16, 48]. In our analysis both postoperative complications as well as the prognostic composite variable were associated with reduced CSS. Whether the application of PRBC is associated with preoperative status (anemia), complications or has an intrinsic effect based on immunosuppressive features might still be up for debate, our data suggests that the avoidance of PRBC might be beneficial for the long-term prognosis of the patient.

Long-term survival and cure emerged as the major treatment goal in cancer, especially from a patient perspective. As mentioned above, papers focusing on long-term survival in pCCA are sparse. In a multicenter study from the United States, Tran et al. analyzed patients undergoing curative-intent surgery from 2000 to 2015 by a multi-institutional registry from 10 U.S. academic medical centers [49]. Here, 257 patients with a 5-year-OS of 19% were investigated. A subgroup of 194 patients deceased or displayed a follow-up longer than 5 years of which 23 (12%) were categorized as long-term survivors. Interestingly, while the 5-year-CSS of our cohort was significantly better with 39% than in the US cohort, the percentages of LTS were comparable (12.9%). However, it has to be noted that the follow-up of this US multicenter study was longer than in our report, making it likely that some of our patient will actually be long-term survivors but did not have enough follow-up time to be classified as such, which would lead to an underestimation of LTS in our cohort. In the US study, most distinct features between LTS and the other patients were the absence of lymph node metastases, R0 resections as well as low CA19-9 values. However, the authors still conclude that 5-year survival can be achieved even in the presence of traditionally unfavorable clinicopathologic factors (elevated CA 19–9, nodal metastasis, and R1 margins). While multicenter studies have the advantage of multiple contributing centers and therefore adjustments for random effects, the dataset is often limited compared to monocentric analyses. As such, it is not surprising that the variables assessed in the US data as nodal metastasis and R1 resections were distinct in the univariate part of our STS versus LTS analysis, but the key differences in the multivariable analysis (PVI and PRBC transfusions) were not part of the data set of the multicenter study.

Given the importance of PVI and PRBC in our STS versus LTS logistic regression analysis and the validation for RFS and CSS of both variables in the Cox regression analysis in the overall cohort, our results can have implications for clinical decision-making. PVI itself is not likely to be modulated by the treating surgeon but should be used for the oncological risk assessment prior to surgery especially if other risk factors e.g. nodal involvement or simultaneous infiltration of the hepatic artery are present. Our results support a detailed tumor staging using state-of-the-art cross sectional to preoperatively determine vessel infiltration [35].

Therefore, any measures of modern patient blood management (PBM) including preoperative optimization, anesthesiologic as well as intraoperative technique and postoperative management should be rigorously applied in patients with pCCA [50]. PBM involves a multidisciplinary approach aimed at optimizing patient outcomes by minimizing the need for allogenic blood transfusions, reducing perioperative bleeding, and managing anemia. Prior to surgery, it is essential to identify and address preexisting anemia while optimizing hemoglobin levels. Proactive management of underlying conditions contributing to perioperative bleeding is imperative. Furthermore, PBM promotes evidence-based transfusion practices, including adherence to restrictive transfusion thresholds based on patient-specific factors rather than arbitrary hemoglobin levels [51]. To mitigate the risk of intraoperative bleeding, potentially necessitating PRBC transfusion, it is advisable to perform these complex surgeries at high-volume, specialized hepatobiliary centers. Intraoperative strategies to minimize bleeding include meticulous surgical techniques and the potential utilization of effective hemostatic agents [52]. Additionally, advanced surgical technologies such as electrocautery and ultrasonic dissection devices (e.g. CUSA) are employed. Whenever feasible, minimally invasive approaches (e.g. robotic surgery for pCCA) are preferred due to their typically reduced blood loss compared to traditional open surgery [53].

Besides the prognostic composite variable, nodal status, postoperative complications exceeding Clavien-Dindo IIIa, reduced hemoglobin, high tumor grading, the presence of LVI and duration of hospitalization have been identified as prognostic variables for impaired oncological outcome in terms of CSS and RFS. These variables display commonly known risk factors in pCCA and indicate comparability with other studies from different workgroups [11, 15, 54].

Concerning lymph node status, a recent retrospective single-center study published in 2023 investigated the prognostic role of lymph node staging in pCCA and was able to demonstrate the prognostic effect of negative lymph node status on long-term survival of up to 5 years [55]. Our work group recently published a retrospective single-center study which clearly determined greater postoperative complications categorized according to Clavien-Dindo scale > 3a to be a negative prognostic predictor for CSS in surgically resected pCCA patientsxs which underlines our multivariate analysis for CSS [56]. The association between tumor size greater than 30 mm and poorer overall survival in pCCA has been historically documented. This relationship may result from the increased likelihood of larger tumors to invade vascular structures, consequently elevating the risk of micrometastasis [57, 58]. These findings could explain our results of a worse RFS in pCCA patients with larger tumors: despite negative resection margins, there is a possibility of scattered tumor cells within the remaining liver tissue due to skip lesions. Tumor differentiation and LVI have been associated to be negative independent predictors for RFS several times [59, 60]. Poor tumor differentiation often suggests a more aggressive and rapidly growing tumor with a higher risk of micrometastasis, which are not detectable by standard imaging methods and thus lead to recurrence later. Another explanation could be a worse response to chemotherapy or other adjuvant therapy strategies in terms of resistance. Lastly, poorly differentiated tumors often exhibit genetic and molecular alterations that promote tumor growth which contributes to the more aggressive behavior and its propensity for recurrence [61]. LVI facilitates the dissemination of cancer cells to distant sites through the lymphatic and blood circulatory systems, increasing the likelihood of metastasis to e.g. regional lymph nodes, serving as a predictor for recurrence of pCCA.

As with all monocentric analyses, this study has several limitations. Since all patients were treated at a single institution, the surgical technique and clinical decision-making reflect the authors individual approach to pCCA which might not be transferable to other centers and datasets. While multivariable analyses were conducted to mitigate confounding effects, it is debatable whether the main prognostic variables PVI and PRBC are influenced by other tumor characteristics. While no re-analyzation of the data might overcome unobserved confounders, reassurance of our findings by independent data sets is of upmost interest to validate our findings in the future. While comprising large data set for a monocentric report, some of the less common clinical features of pCCA can still not proficiently be analyzed. This particularly accounts for neoadjuvant therapy which was rarely applied in our cohort. Also, some pathological features, which have been reported to be prognostic (e.g. PNI), might have a more pronounced effect in a larger data set. Unfortunately, the data availability regarding the tumor marker CA 19–9 was limited, so it was not included in the multivariate analysis.

Despite the above-mentioned limitations, we were able to demonstrate that a notable fraction of patients with pCCA might achieve LTS. The combination of PVI and PRBC transfusions has been identified as an independent prognostic factor for dismal oncological outcome in pCCA patients. These results emphasize the possibility for further patient selection prior to surgery and stress the importance of an optimized clinical and perioperative management.

## Supporting information

S1 Table Logistic regression for intraoperative PRBC transfusion. Various parameters are associated with PRBC transfusion. ALT, alanine aminotransferase; ASA, American society of anesthesiologists classification; AST, aspartate aminotransferase; BMI, body mass index; CRP, c-reactive protein; EBD, endoscopic biliary drainage; FFP, fresh frozen plasma; GGT, gamma glutamyltransferase; ICU, intensive care unit; INR, international normalized ratio; LVI, lympho-vascular invasion; OS, overall survival; MVI, microvascular invasion; PBD, percutaneous biliary drainage; PNI, perineural invasion; PVE, portal vein embolization.
(DOCX)

## Abbreviations

9: Carbohydrate antigen 19–9
ALPPS: Associating liver partition and portal vein ligation for staged hepatectomy
ALT: Alanine aminotransferase
ASA: American society of anesthesiologists
AST: Aspartate aminotransferase
BMI: Body mass index
CCA: Cholangiocellular carcinoma
CI: Confidence interval
CRP: C reactive protein
CSS: Cancer-specific survival
CT: Computed tomography
CUSA: Cavitron Ultrasonic Surgical Aspirator
dCCA: Distal cholangiocarcinoma
EBD: Endoscopic biliary drainage
ERCP: Endoscopic retrograde cholangiopancreatography
FFP: Fresh frozen plasma
FLR: Future liver remnant
GGT: Gamma glutamyltransferase
iCCA: Intrahepatic cholangiocarcinoma
INR: International normalized ratio
LiMAx: Maximum liver function capacity
LTS: Long-term survivors
MRCP: Magnetic resonance cholangiopancreatography
MRI: Magnetic resonance imaging
OS: Overall Survival
PBD: Percutaneous biliary drainage
PBM: Patient blood management
pCCA: Perihilar cholangiocarcinoma
PRBC: Packed red blood cells
PV: Portal vein
PVE: Portal vein embolization
PVI: Portal vein involvement
PVO: Portal vein occlusion
RFS: Recurrence-free survival
RWTH: Rheinisch-Westfälische Technische Hochschule
STS: Short-term survivors
UICC: Union for international cancer control
