# Supplementary material for: Clinical features and prediction of long-term survival after surgery for perihilar cholangiocarcinoma
Source: PLoS One. 2024 Jul 1;19(7):e0304838. doi: 10.1371/journal.pone.0304838 (PMC11216605; doi:10.1371/journal.pone.0304838)
Supplement: S1 Table — Various parameters are associated with PRBC transfusion. ALT, alanine aminotransferase; ASA, American society of anesthesiologists classification; AST, aspartate aminotransferase; BMI, body mass index; CRP, c-reactive protein; EBD, endoscopic biliary drainage; FFP, fresh frozen plasma; GGT, gamma glutamyltransferase; ICU, intensive care unit; INR, international normalized ratio; LVI, lympho-vascular invasion; OS, overall survival; MVI, microvascular invasion; PBD, percutaneous biliary drainage; PNI, perineural invasion; PVE, portal vein embolization. (DOCX) [file pone.0304838.s001.docx]

**S1 Table. Logistic regression for intraoperative PRBC transfusion**

|  | **Univariate analysis** | |  | **Multivariate analysis** | |
| --- | --- | --- | --- | --- | --- |
|  | **HR (95% CI)** | ***P* value** |  | **HR (95% CI)** | ***P* value** |
| **Demographics** |  |  |  |  |  |
| Sex (male=1) | 1.43 (0.80 – 2.55) | .223 |  |  |  |
| Age (≤ 65 years=1) | 1.35 (0.78 – 2.33) | .289 |  |  |  |
| BMI (≤ 25 kg/m^2^=1) | 0.84 (0.49 – 1.45) | .535 |  |  |  |
| Bismuth type (I/II=1) | 1.05 (0.57 – 1.93) | .876 |  |  |  |
| Neoadjuvant therapy (no=1) | 2.45 (0.61 – 9.76) | .203 |  |  |  |
| PVE (no=1) | 0.55 (0.31 – 0.97) | .**039** |  |  | .175 |
| ASA (I/II=1) | 0.95 (0.54 – 1.65) | .844 |  |  |  |
| Preoperative cholangitis (no=1) | 0.46 (0.25 – 0.84) | **.011** |  |  | .452 |
| EBD (no=1) | 0.88 (0.48 – 1.61) | .676 |  |  |  |
| PBD (no=1) | 0.27 (0.13 – 0.53) | .**000** |  | 0.22 (0.07 – 0.66) | .**007** |
| Portal vein infiltration > 180° (no=1) | 1.47 (0.85 – 2.55) | .168 |  |  |  |
| Arterial infiltration > 180° (no=1) | 3.19 (1.49 – 6.83) | **.003** |  | 4.11 (1.03 – 16.50) | **.046** |
| Lobar atrophy (no=1) | 0.94 (0.52 – 1.70) | .831 |  |  |  |
| sFLR (≤ 40%=1) | 0.85 (0.45 – 1.62) | .626 |  |  |  |
| **Clinical chemistry** |  |  |  |  |  |
| Albumin (≤ 35 g/l=1) | 0.39 (0.21 – 0.72) | .**003** |  |  | .564 |
| AST (≤ 50 U/l=1) | 0.74 (0.43 – 1.30) | .294 |  |  |  |
| ALT (≤ 50 U/l=1) | 0.64 (0.35 – 1.19) | .158 |  |  |  |
| GGT (≤ 400 U/l=1) | 0.71 (0.41 – 1.24) | .232 |  |  |  |
| Bilirubin (≤ 1 mg/dl=1) | 1.15 (0.67 – 1.98) | .623 |  |  |  |
| Alkaline phosphatase (≤ 250 U/l=1) | 1.15 (0.66 – 2.01) | .616 |  |  |  |
| Platelet count (≤ 300 /nl=1) | 1.12 (0.65 – 1.93) | .684 |  |  |  |
| INR (≤ 1=1) | 1.32 (0.75 – 2.33) | .338 |  |  |  |
| Hemoglobin (≤ 12 g/dl=1) | 0.21 (0.12 – 0.38) | **.000** |  | 0.12 (0.04 – 0.36) | **.001** |
| CRP, mg/l (≤ 10 mg/l=1) | 1.46 (0.84 – 2.55) | .184 |  |  |  |
| **Operative data** |  |  |  |  |  |
| Operative time (≤ 360 min=1) | 1.66 (0.95– 2.90) | .076 |  |  |  |
| Type of resection |  | .**006** |  |  | .298 |
| Right/Left hepatectomy | 1 |  |  |  |  |
| Extended hepatectomy | 2.31 (1.27 – 4.21) |  |  |  |  |
| Hepatoduodenectomy | 1.67 (0.53 – 5.27) | .385 |  |  |  |
| Vascular resection (no=1) | 3.29 (1.03– 10.57) | .045 |  |  |  |
| FFP transfusion (no=1) | 12.30 (6.36 – 23.78) | **.000** |  | 22.63 (7.32 – 69.97) | **.001** |
| **Pathological data** |  |  |  |  |  |
| R1 resection (no=1) | 1.86 (0.91 – 3.77) | .087 |  |  |  |
| pT category (T1/T2=1) | 1.48 (0.83 – 2.65) | .187 |  |  |  |
| Tumorsize (≤ 30 mm=1) | 2.27 (1.28– 4.02) | **.005** |  |  | .510 |
| pN category (N0=1) | 1.48 (0.85 – 2.56) | .163 |  |  |  |
| Tumor grading (G1/G2=1) | 1.44 (0.75– 2.74) | .269 |  |  |  |
| MVI (no=1) | 1.45 (0.80– 2.65) | .223 |  |  |  |
| LVI (no=1) | 1.15 (0.60 – 2.22) | .680 |  |  |  |
| PNI (no=1) | 1.62 (0.76– 3.44) | .214 |  |  |  |
| **Postoperative data** |  |  |  |  |  |
| ICU time (≤ 1 days=1) | 2.74 (1.60 – 4.81) | **.000** |  |  | .481 |
| Hospitalization (≤ 21 days=1) | 3.40 (2.12 – 7.54) | **.000** |  | 2.92 (1.14 – 7.49) | **.001** |
| Complications (≤ 3a=1) | 2.70 (1.52 – 4.78) | **.001** |  |  | .219 |
| **Oncological data** |  |  |  |  |  |
| Adjuvant therapy (no=1) | 0.89 (0.48 – 1.64) | .703 |  |  |  |

*Various parameters are associated with PRBC transfusion. ALT, alanine aminotransferase; ASA, American society of anesthesiologists classification; AST, aspartate aminotransferase; BMI, body mass index; CRP, c-reactive protein; EBD, endoscopic biliary drainage; FFP, fresh frozen plasma; GGT, gamma glutamyltransferase; ICU, intensive care unit; INR, international normalized ratio; LVI, lympho-vascular invasion; MVI, microvascular invasion; PBD, percutaneous biliary drainage; PNI, perineural invasion; PVE, portal vein embolization*
